# Fully Compressible Low-Mach Number Simulations of Carbon-dioxide at Supercritical Pressures and Trans-critical Temperatures

**DOI:** 10.1007/s10494-017-9872-4

**Published:** 2017-11-21

**Authors:** Uttiya Sengupta, Hassan Nemati, Bendiks J. Boersma, Rene Pecnik

**Affiliations:** Department of Process and Energy, Delft University of Technology, Leeghwaterstraat 39, 2628 CB Delft, Netherlands

**Keywords:** Direct numerical simulation, Supercritical fluid, Trans-critical transition, Peng Robinson equation, Solenoidal dissipation, Extended van Driest transformation

## Abstract

This work investigates fully developed turbulent flows of carbon-dioxide close to its vapour-liquid critical point in a channel with a hot and a cold wall. Two direct numerical simulations are performed at low Mach numbers, with the trans-critical transition near the channel centre and the cold wall, respectively. An additional simulation with constant transport properties is used to selectively investigate the effect of the non-linear equation of state on turbulence. Compared to the case where the pseudo-critical transition occurs in the channel center, the case with the pseudo-critical transition close to the cold wall reveals that compressibility effects can exist in the near-wall region even at low Mach numbers. An analysis of the velocity streaks near the hot and the cold walls also indicates a greater degree of streak coherence near the cold wall. A comparison between the constant and variable viscosity cases at the same Reynolds number, Mach number and having the same isothermal wall boundary conditions reveals that variable viscosity increases turbulence near the cold wall and also causes higher velocity gradients near the hot wall. We also show that the extended van Driest transformation results in a better agreement of the velocity profile with the log-law of the wall compared to the standard van Driest transformation. The semi-locally scaled turbulent velocity fluctuations and the turbulent kinetic energy budgets on the hot and the cold sides of the channel collapse on top of each other, thereby establishing the validity of Morkovin’s hypothesis.

## Introduction

**Fig. 1:**
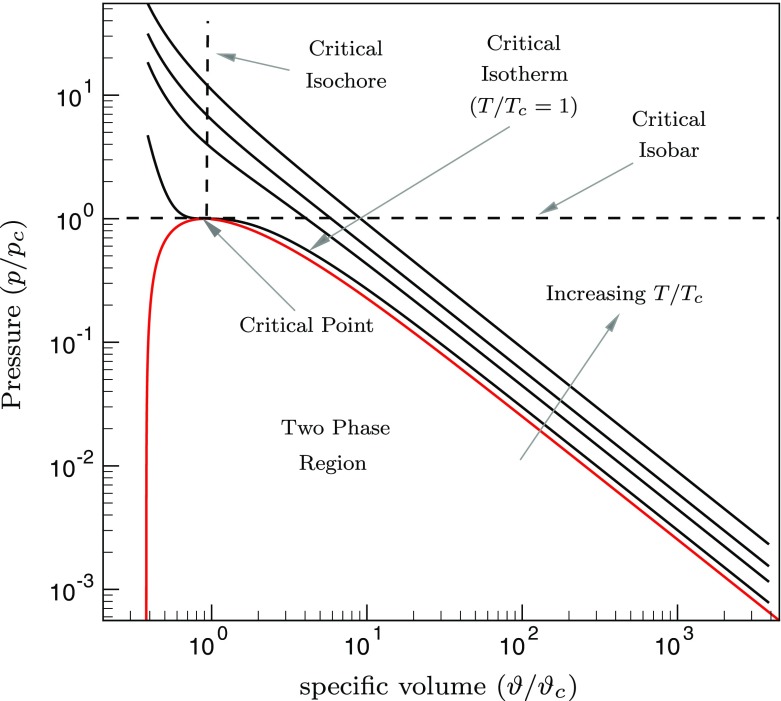
Pressure as a function of specific volume normalized by their critical values at constant temperatures for CO_2_;  *T*/*T*_*c*_ =1.0;*T*/*T*_*c*_=1.5, *T*/*T*_*c*_=2.0, *T*/*T*_*c*_=3.0;  saturation lines

**Fig. 2:**
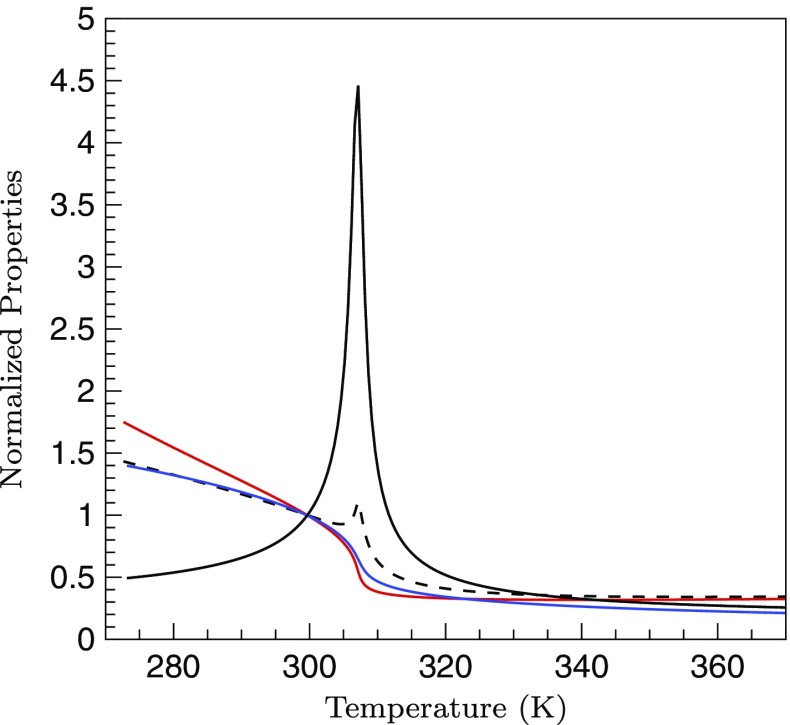
Thermodynamic and transport properties of CO_2_ normalized by their values at the reference conditions as a function of temperature at a pressure of 80 bar.  heat capacity (*C*_*p*_/*C*_*p*0_); density (*ρ*/*ρ*_0_);  viscosity (*μ*/*μ*_0_);  thermal conductivity (*κ*/*κ*_0_)

Fluids at temperatures and pressures above their vapour-liquid critical point are known as supercritical fluids. Above the vapour-liquid critical point, the heat capacity of a supercritical fluid at constant pressure (*C*_*p*_) shows a maximum value at a certain temperature, known as the pseudo-critical temperature (*T*_*pc*_). This point is called the pseudo-critical point. The line joining the pseudo-critical points for a range of pressures above the critical point is called the pseudo-critical line. Below the critical point, the fluid exists as a mixture of liquid and vapour in the two phase region. The line separating the supercritical fluid from the two phase region consists of the saturation liquid and the saturation vapour lines. Very near to the critical point, the fluid can neither be called a liquid nor a gas and has a unique ombination of gas-like and liquid-like properties. The area bounded by the critical isobar and the critical isotherm represents supercritical fluids, as shown in Fig. 1. The pressure and specific volume are normalized by the critical pressure (*p*_*c*_) and the critical volume (*v*_*c*_) of CO_2_, respectively. Along any isobar above the critical point, on crossing the pseudo-critical point, all the thermodynamic and transport properties like density, isobaric heat capacity, viscosity and thermal conductivity etc., exhibit sharp gradients for small changes in temperature. This phenomena is called trans-critical transition. This has been shown in Fig. 2 by plotting the density (*ρ*), heat capacity (*C*_*p*_), viscosity (*μ*) and the thermal conductivity (*κ*) for supercritical CO_2_ at 80 bar as a function of temperature, normalized by their values at the reference condition of 80 bar and 300 K. The reference values for these properties are mentioned in Table 1. The density and the heat capacity are calculated from the Peng Robinson equation of state and the viscosity and the thermal conductivity are calculated using the Refprop library [1]. Due to these unique characteristics, supercritical fluids have been the subject of research for a considerable period of time. These fluids have various applications in the industry, such as in supercritical power cycles, in drug delivery systems, as solvents in the extraction of plant materials and also as solvents in the extraction of polymers.

**Table 1:** Density (*ρ*_0_), Heat capacity (*C*_*p*0_), viscosity (*μ*_0_) and thermal conductivity (*κ*_0_) for supercritical CO_2_ under the reference conditions of 80 bar and 300 K

*ρ*_0_	*C*_*p*0_	*μ*_0_	*κ*_0_
689.46 kg/m^3^	4.98 kJ/kgK	6.093 ∗ 10^−5^ Pa.s	8.246 ∗ 10^−2^ W/mK

In many of the engineering applications of supercritical fluids mentioned above, buoyancy can play a role. Our simulations, however, have not taken into account the effects of buoyancy. A discussion is henceforth provided to include some of the effects of buoyancy in turbulent flows. Publications by previous authors, such as, [2, 3] have discussed in detail the changes in the nature of turbulence in channel flows and also in Poiseuille-Rayleigh-Bernard (PRB) flows due to buoyancy. This involved performing DNS under the assumption of temperature independent fluid properties, i.e. under the Oberbeck-Boussinesq hypothesis (OB) and comparing the results to simulations with viscosity (*μ*) and thermal expansion coefficient (*β*) varying with temperature. In case of PRB flows, the wall-normal transport of momentum and heat is increased by buoyancy compared to the case of forced convection where temperature is a passive scalar and the mixing efficiency increases with the increase in Richardson number (*R* *i*_*τ*_). In case of a stable stratified flow in a channel with a top hot wall, a bottom cold wall and gravity acting downward in the wall-normal direction, it is seen that the fluid particles near the hot wall experience a larger buoyancy force resulting in a partial laminarization of the flow near the hot wall. If the location of the hot and cold walls are reversed, then it would result in unstable stratification. This is in contrast to forced convection flows, where only the wall temperatures are significant and the location of the hot and cold wall on the bottom or the top are irrelevant.

In case of turbulent flows of supercritical fluids, it is found that the sharp property variations alter the conventional behaviour of gas dynamics and turbulence. Direct numerical simulations for upward annular flows of supercritical CO_2_ with constant heat-flux wall boundary conditions was conducted by [4]. It was found that the most singular phenomenon is observed at supercritical pressures when there is trans-critical transition in the flow. This leads to some peculiar characteristics in the turbulent flow field, such as heat transfer deterioration causing the weakening of the velocity streaks near the point of the maxima of temperature fluctuation and also, the weakening of ejection and sweep motions near the wall. Large eddy simulation (LES) of a turbulent jet of nitrogen under supercritical pressure was performed by [5]. It compares low pressure LES simulations and real gas thermodynamics with data extracted from experiments. This approach has indicated that the high density gradients and non-linear thermodynamics has a limited impact on the jet spreading rate and the pseudo-similarity behaviour for jets. Turbulence models are still not capable of providing satisfactory results for wall-bounded turbulent flows with strong heat transfer, as shown in [6]. A review of the turbulent flows of supercritical fluids was conducted by [7] in order to study various effects like buoyancy, flow acceleration and heat transfer deterioration in supercritical fluid flows. A numerical method has however been provided by [8] for conducting high fidelity simulations for supercritical fluids involving trans-critical transition. [9] has investigated developing turbulent flows of supercritical CO_2_ in a pipe geometry involving forced and mixed convection. The effects of flow stratification caused by buoyancy for turbulent flows of supercritical CO_2_ in a pipe have been investigated by [10]. Large eddy simulations of cryogenic nitrogen injection have been performed by [11] using the Peng Robinson equation of state at supercritical pressures. In this paper, various subgrid-scale (SGS) models have been compared and the influence of the density gradients on the growth of instability have been investigated. One dimensional rarefraction properties in compression shocks have been investigated recently by [12] for non-ideal gases close to the vapour-liquid critical point. [13] also investigates the heat transfer characteristics in fully developed turbulent flows of CO_2_ in an annular geometry by simulating the low-Mach number approximation Navier-Stokes equations. In many previous works, such as [4, 7, 9] and [13], Navier-Stokes equations have been solved using the low-Mach number approximation, which takes into account changes in density due to changes in temperature, but ignores the effect of density changes due to the pressure variations. In order to further improve our understanding of the fluid dynamics of supercritical fluids and also to capture the compressibility effects, a fully compressible Navier-Stokes solver has been developed, which can include the effects of density changes due to the variation of both pressure and temperature. Cases have been simulated with isothermal wall boundary conditions such that we get trans-critical transition inside the flow field. Simulations with constant and variable transport properties have been compared in order to estimate the effects of variable viscosity. The effects of the changing location of trans-critical transition have been investigated by simulating a flow with *T*_*pc*_ close to the cold wall.

This paper mainly analyzes the influence of trans-critical transition on near-wall turbulence and compressibility effects in fully developed turbulent flows of supercritical CO_2_ near the critical point at low Mach numbers. It further investigates the validity of scaling methodologies, such as the extended van Driest velocity scaling and the semi-local scaling for these flows. It is organized as follows: Section 2 shows the non-dimensionalized form of the compressible Navier-Stokes equations; Section 3 gives the details of various thermodynamic equations of state including the rationale for choosing Peng Robinson as the preferred equation of state, and the transport properties; Section 4 gives the numerical methods and simulation details; Section 5 gives the results and discussions; Section 6 gives the conclusions.

## Navier-Stokes Equations

An in-house code has been developed to solve the compressible Navier-Stokes equations in Cartesian coordinates for fully developed turbulent flow in a channel. The continuity, momentum and energy equations are solved for the density (*ρ*), the components of momentum in three directions (*ρ* *u*_*i*_) and the total energy (*ρ* *e*_0_), respectively. The total energy is expressed as $\rho e_{0} = \rho e_{int} +1/2 \rho {u_{i}^{2}}$, where *e*_*int*_ is the internal energy and $1/2 \rho {u_{i}^{2}}$ is the kinetic energy. The non-dimensional Navier-Stokes equations are given as follows,

1 $$ \frac{\partial \rho}{\partial t} + \frac{\partial \rho u_{j}}{\partial x_{j}} =0, $$

2 $$ \frac{\partial \rho u_{i}}{\partial t} + \frac{\partial \left( \rho u_{i} u_{j} + p\delta_{ij} - {\Gamma}_{ij}\right)}{\partial x_{j}} = \rho f_{i}, $$

3 $$ \frac{\partial \rho e_{0}}{\partial t} + \frac{\partial \left( u_{j}\rho e_{0} + u_{j}p + q_{j} - u_{i}{\Gamma}_{ij}\right)}{\partial x_{j}} = \rho u_{j} f_{j}, $$

with the viscous stress Γ_*ij*_and *q*_*j*_the heat flux defined as,

4 $$ {\Gamma}_{ij} = \frac{\mu}{Re}\left[\left( \frac{\partial u_{i}}{\partial x_{j}} + \frac{\partial u_{j}}{\partial x_{i}}\right) - \frac{2}{3}\delta_{ij} \frac{\partial u_{k}}{\partial x_{k}}\right], $$

5 $$ q_{j} = - \frac{\kappa} {Re Pr M_{ps}^{2}}\frac{\partial T}{\partial x_{j}}. $$

In the above, *μ* is the viscosity, *p* denotes the pressure, *κ* is the thermal conductivity and *f*_*i*_is the applied forcing term in streamwise direction for the conservation of mass flux. The applied forcing term is analogous to (−1/*ρ*)*∂* *p*/*∂* *x* for pressure driven flows and is calculated in a way such that the quantities $\int \limits \rho dy$ and $\int \limits \rho U dy$ are conserved. The symbol *U* refers to the velocity in the streamwise direction. The constant integrated density follows directly as a result of the continuity equation and the applied pressure gradient results in the conservation of the streamwise momentum. The non-dimensionalization is performed as follows.

$$x_{i} = \frac{x_{i}^{\star}}{H^{\star}},\quad t = \frac{t^{\star} U_{b}^{\star}}{H^{\star}},\quad e_{0} = \frac{e_{0}^{\star}}{U_{b}^{\star 2}},\quad u_{i} = \frac{u_{i}^{\star}}{U_{b}^{\star}}, $$

6 $$ p = \frac{p^{\star}}{\rho_{0}^{\star} U_{b}^{\star 2}}, \quad \rho = \frac{\rho^{\star}}{\rho_{0}^{\star}},\quad \kappa = \frac{\kappa^{\star}}{\kappa_{0}^{\star}},\quad T = \frac{T^{\star}}{T_{0}^{\star}},\quad \mu = \frac{\mu^{\star}}{\mu_{0}^{\star}}. $$

The superscript ⋆is used to denote dimensional quantities. The non-dimensional numbers, namely, the Reynolds number, Prandtl number and Mach number are given as,

7 $$ Re = \frac{\rho_{0}^{\star} U_{b}^{\star} h^{\star}}{\mu_{0}^{\star}},\quad Pr = \frac{\mu_{0}^{\star} C_{p0}^{\star}}{ \kappa_{0}^{\star}},\quad M = \frac{U_{b}^{\star}}{C_{0}^{\star}}, $$

where $C_{0}^{\star }$ is the speed of sound under the reference conditions. The pseudo-Mach number used to non-dimensionalize the heat conduction term is given as

8 $$ M_{ps}^{2} = \frac{U_{b}^{\star 2}}{C_{p0}^{\star}T_{0}^{\star}}. $$

The subscript 0 is used to denote the properties of CO_2_ under the reference conditions of 80 bar and 300 K.

## Thermodynamic Equations of State and Transport Properties

### Equation of state

The choice of the equation of state is important as it is essential to accurately represent the thermodynamic properties of CO_2_ near the critical point in a computationally efficient manner. The equation of state can either be implemented directly in the Navier-Stokes solver or a lookup table approach can be used to tabulate the required thermodynamic properties that can then be obtained using an appropriate interpolation method. The thermodynamic properties, such as, pressure and temperature have to be evaluated in terms of the conservative variables density and internal energy. When the equation of state is implemented directly in the Navier-Stokes solver, temperature and pressure are evaluated by solving the departure function for internal energy by using a Newton-Raphson solver. Multiparameter equations of state, such as, [14] and [15] have a high level of accuracy near the critical point of CO_2_. However, these equations of state involve complicated terms and implementing the Newton-Raphson solver can be computationally very expensive. The lookup table approach suffers from the problem of consistency as normal interpolation methods are not consistent upto the 2^nd^ order derivative for these properties, as mentioned by [16]. In order to reach a compromise between accuracy, computational efficiency and consistency, a cubic equation of state is used, which is not as accurate as the multiparameter equations but is computationally more efficient. It also does not suffer from the problem of consistency like the lookup table approach. The Peng Robinson equation of state has been selected after careful consideration.

The cubic equation of state (EOS) to be used is chosen by comparing the accuracy of the thermodynamic properties predicted by the different cubic equations near the critical point of CO_2_. The thermodynamic properties like density and heat capacity (*C*_*p*_) predicted by the van der Waals (VdW), Peng Robinson (PR) and Redlich Kwong (RK) equation of state at constant pressure of 80 bar are compared with the actual values extracted from the Refprop library.

In order to calculate thermodynamic properties from a real gas, the departure function for that property with respect to the chosen equation of state has to be evaluated. The departure function for any thermodynamic property of a real gas is defined as the difference in the value of that property determined from the chosen real gas equation of state and the value of the same property for an ideal gas under the same conditions of temperature and pressure. Mathematically, it can be expressed as follows,

9 $$ X^{R}\left( T,P\right) = X\left( T,P\right) - X_{ig}\left( T,P\right), $$

where *X*(*T*,*P*)is a thermodynamic property of the real gas at temperature (*T*) and pressure (*P*), *X*_*ig*_(*T*,*P*) is the same property for the ideal gas (IG) and *X* ^*R*^(*T*,*P*)is the departure function under the same conditions of temperature and pressure. For the compressible Navier-Stokes solver, the energy equation calculates the total energy. The kinetic energy is then subtracted from the total energy to determine the internal energy. The calculation of departure functions for the internal energy from the van der Waals and the Redlich Kwong equation of state is mentioned in Appendix A. The departure function for the Peng Robinson equation of state is derived as follows.

The non-dimensional Peng Robinson equation of state is given as

10 $$ P = \frac{RT}{\vartheta - b} - \frac{a\alpha}{\vartheta{^{2}} +2b\vartheta - b^{2}}, $$

where the non-dimensionalization is performed by

11 $$ R=\frac{R^{\star} T_{0}^{\star}}{U_{0}^{\star 2}};a=\frac{a^{\star} \rho_{0}^{\star}}{U_{0}^{\star 2}};b=b^{\star}\rho_{0}^{\star}, $$

with *𝜗* =1/*ρ*, $a^{\star } =0.457235 R^{\star 2} T_{c}^{\star 2} / P_{c}^{\star }$ and $b^{\star } =0.077796 R^{\star } T_{c}^{\star }/P_{c}^{\star }$. The parameter *α* is determined by the acentric factor *ω* and the reduced temperature *T*_*r*_ = *T*/*T*_*c*_as,

12 $$ \alpha = \left( 1 + K\left( 1 - T_{r}^{0.5}\right)\right)^{2}, K = 0.37464 + 1.54226\omega - 0.26992\omega^{2}. $$

The residual internal energy, *U* ^*R*^, is given as:

13 $$ U^{R} = U - U^{ig} = \int\limits_{\infty}^{\vartheta}\left( T\left( \frac{\partial S}{\partial \vartheta}\right)_{T} - P\right)d\vartheta. $$

Substituting Maxwell’s relations, (*∂* *S*/*∂* *𝜗*)_*T*_ =(*∂* *P*/*∂* *T*)_*𝜗*_, in Eq. 13 we obtain

14 $$ U^{R} = \int\limits_{\infty}^{\vartheta}\left[T\left( \frac{\partial P}{\partial T}\right)_{\vartheta} - P\right]d\vartheta, $$

15 $$ U - C_{\vartheta}T = \int\limits_{\infty}^{\vartheta} \frac{a\left( \alpha - T\frac{d \alpha}{d T}\right)}{2\sqrt{2}b}\left[\frac{1}{\left( \vartheta + b -\sqrt{2}b\right)} - \frac{1}{\left( \vartheta + b +\sqrt{2}b\right)}\right] $$

16 $$ U = C_{\vartheta}T + \frac{a\left( \alpha - T\frac{d \alpha}{d T}\right)}{2\sqrt{2}b} log \frac{\left( 1 + b\left( 1 -\sqrt{2}\right)\rho\right)}{\left( 1 + b\left( 1 +\sqrt{2}\right)\rho\right)}. $$

**Fig. 3:**
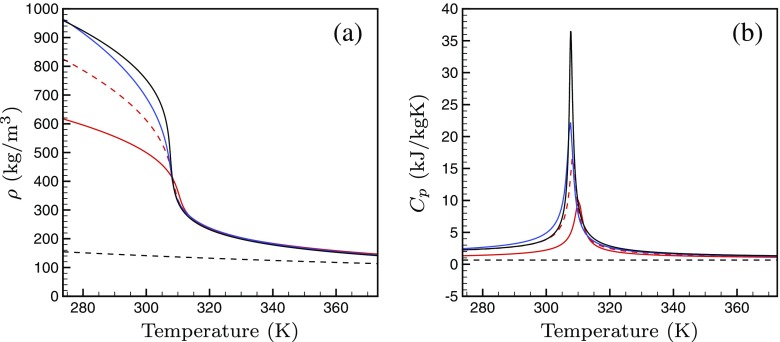
Density as a function of temperature for supercritical CO_2_ at 80 bar for different cubic equations of state compared to that from the Refprop library [1].  IG, VdW, RK, PR, Refprop

The enthalpy is related to internal energy as *H* ^0^ = *U* + *P*/*ρ* and the heat capacity at constant pressure is *C*_*p*_ =(*∂* *H* ^0^/*∂* *T*)_*p*_. The plots in Fig. 3a and b showing the density and the heat capacity at constant pressure (*C*_*p*_) for supercritical CO_2_ at 80 bar as a function of temperature, establish that the Peng Robinson equation of state represents the actual properties obtained from the Refprop library far more accurately than the other cubic equations of state, such as van der Waals equation and the Redlich Kwong equation of state. Hence, the Peng Robinson equation is chosen to calculate the thermodynamic properties in the Navier-Stokes solver.

### Implementation of the Peng Robinson equation

The non-dimensionalized departure function for the Peng Robinson equation of state is given as

17 $$ U = \frac{R T_{r} T_{c}} {\gamma - 1} + \frac{\left( 1+K\right)\left( 1+K\left( 1-T_{r}^{0.5}\right)\right)a}{2\sqrt{2}b} log \left( \frac{1+b\left( 1-\sqrt{2}\right)\rho}{1+b\left( 1+\sqrt{2}\right)\rho}\right), $$

where *γ* = *R*/*C*_*𝜗*_ +1, and *T*_*r*_ = *T*/*T*_*c*_ is the reduced non-dimensional temperature. It is evident that the equation above is quadratic in $\sqrt {T_{r}}$. It can be represented as

18 $$ A \sqrt{T_{r}}^{2} + B \sqrt{T_{r}} + C = 0, $$

where *A*,*B* and *C* are

19 $$ A = \frac{RT_{c}}{\gamma-1}, $$

20 $$ B = - \frac{K\left( 1+K\right)a}{2\sqrt{2}b}log\left( \frac{1+b\left( 1-\sqrt{2}\right)\rho}{1+b\left( 1+\sqrt{2}\right)\rho}\right), $$

21 $$ C = \frac{\left( 1+K\right)^{2}a}{2\sqrt{2}b}log\left( \frac{1+b\left( 1-\sqrt{2}\right)\rho}{1+b\left( 1+\sqrt{2}\right)\rho}\right) - U . $$

The analytical formula for finding the roots of a quadratic equation is used to determine the roots. Two positive roots are not possible for this equation as it is thermodynamically not possible to have the same internal energy for a gas at a particular density and two different temperatures. Therefore, one of the roots is positive and the other is negative. The negative root is frivolous and is neglected. We can thus arrive at a unique solution for the temperature. This methodology is adopted to avoid iterations with a Newton-Raphson solver and to arrive at the solution in a computationally efficient manner.

### Transport properties

The Refprop library calculates transport properties, such as viscosity and thermal conductivity using the transport models developed by [17, 18]. Instead of directly calling Refprop, the transport properties are tabulated within a relevant range for temperature and pressure and are then stored in a lookup table. For any point inside the flow domain, transport properties like viscosity and thermal conductivity are uniquely determined at each point by calculating the indices in the lookup table and using a 2^*nd*^ order bilinear interpolation method to interpolate between the closest points. The lookup table approach for calculating the transport properties is not computationally expensive, as it involves a cartesian table in temperature and pressure, which are previously calculated using the Peng Robinson equation of state. As temperature and pressure are both known inputs to the table, the indices can be calculated directly without employing a search algorithm.

## Numerical Methods and Simulated Cases

**Fig. 4:**
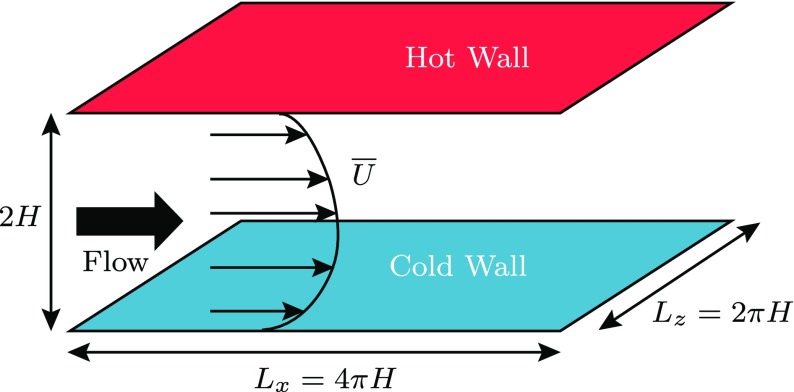
Flow geometry for fully developed turbulent channel flow with wall temperature difference

The turbulent channel flow geometry is given in Fig. 4. The channel is periodic in streamwise and spanwise directions with streamwise length *L*_*x*_ =4*π* *H*, spanwise length *L*_*z*_ =2*π* *H* and wall normal height *L*_*y*_ =2*H*, where *H* is the half channel height. The number of grid points used is 720 × 720 × 360 in the streamwise, spanwise and wall-normal directions, respectively. In streamwise and spanwise directions, the mesh is uniform and pseudo spectral methods are used to calculate the derivatives using a Fast Fourier Transform (FFT) library. In order to minimize aliasing errors, the derivatives for the advective terms in the periodic directions are calculated using the quasi skew-symmetric method developed by [19]. In the wall-normal direction, the mesh is fully collocated and a finite volume scheme is used in which the derivatives for the advective and the diffusive terms are obtained by interpolating the quantities to the cell faces and then differentiating to obtain the values of the derivatives at the cell center. The interpolation and differentiation near the center of the channel are performed in accordance with the 6^*th*^ order compact finite difference method developed by [20] for collocated meshes. 4^*th*^ order boundary schemes also developed by [20] are used for implementing the Dirichlet boundary condition for temperature and velocity at the wall. In the course of our simulations, it is not necessary to calculate the density and pressure at the wall. In the energy equation, the pressure is multiplied with the velocity and the product vanishes at the walls due to the no slip boundary condition. However, the wall-normal gradient of pressure at the walls is required in the wall-normal momentum equation, which is set to zero as an approximation. The terms involving density in the continuity, momentum and energy equations (*ρ* *u*_*i*_, *ρ* *u*_*i*_ *u*_*j*_, *ρ* *u*_*j*_ *e*_0_) are always multiplied with velocities and therefore, the value of these terms at the walls is also equal to zero. For these terms, the 4^th^ order scheme for Dirichlet boundary conditions, developed by [20] can be used without calculating the pressure and density at the walls. The time integration is performed using an explicit third order Runge Kutta method as given by [21]. A hyperbolic tangent function is used to obtain a non-uniform mesh in the wall-normal direction. The domain is parallelized using 2DECOMP&FFT library with 2D pencil decomposition. The code has been validated for fully developed turbulent flows of an ideal gas with the data published by [22] and [23]. This is shown in Appendix B.

Three cases are simulated which have a bulk Reynolds Number of 2800 (based on the reference conditions, bulk velocity and half channel height), bulk pressure (*P*_0_) of 80 bar, reference temperature (*T*_0_) of 300 K, and a reference Mach number of 0.2. All simulations use the Peng Robinson equation of state to calculate the temperature and pressure. The Prandtl number at reference conditions is 3.019 and the speed of sound is 261.4 m/s. All the simulations are forced convection flows without the influence of gravity.

The differences between the three cases are highlighted in Table 2. The first two cases have variable transport properties tabulated from Refprop and are called supercritical variable property cases SC_1_ and SC_2_, respectively. The third case has constant viscosity and thermal conductivity calculated at the reference conditions and is called the supercritical constant transport property (SCCTP) case. The cases SC_1_ and SCCTP have isothermal wall boundary conditions with temperatures 300 K and 315 K at the two walls. For the case SC_2_, the wall temperatures are 306 K and 321 K. At a pressure of 80 bar for carbon dioxide, the pseudo-critical temperature of CO_2_ is around 307.5 K. Thus, the cases SC_1_ and SCCTP have the trans-critical transition roughly between the middle of the channel and the hot wall, whereas the case SC_2_ has trans-critical transition very close to the cold wall.

**Table 2:** Details of the simulations performed using supercritical CO_2_, stating cold and hot wall temperatures, *T*_*wc*_,*T*_*wh*_, viscosity and thermal conductivity, *μ*,*κ*, and resulting friction Reynolds number at both walls

Case	*T*_*wc*_, *T*_*wh*_	Transport properties *μ*,*κ*	*R* *e*_*τwc*_	*R* *e*_*τwh*_
SC_1_	300 K, 315 K	Tabulated Refprop data	218.18	310.12
SC_2_	306 K, 321 K	Tabulated Refprop data	343.63	438.52
SCCTP	300 K, 315 K	Constant	126.41	224.46

Table 3 reports mesh resolutions for all cases in terms of Kolmogorov and Batchelor scales. The Kolmogorov length scales, which indicate the resolution of the momentum scales are defined as $\eta = \sqrt [4]{\left (\left (\overline {\mu } / \overline {\rho }\right )^{3} \overline {\rho } / \epsilon \right )} $. Here, *μ*, *ρ* and *𝜖* refer to the viscosity, density and the dissipation of turbulent kinetic energy obtained from the turbulent kinetic energy budgets of the simulations, respectively. As the Prandtl number in supercritical fluids is more than unity, the mesh resolution in terms of Batchelor scales is also important as the Batchelor scales indicate the resolution of the thermal scales. The Batchelor scales are defined as: $\eta _{B} = \eta / \sqrt {Pr}$, where Pr refers to the Prandtl number. The maximum mesh resolution in terms of the Kolmogorov length scales are within the limits Δ*y* <2*η*, Δ*z* <6*η* and Δ*x* <12*η*, as specified by previous authors [24, 25].

**Table 3:** Spatial resolution with respect to Kolmogorov scales (*η*) and Batchelor scales (*η*_*B*_) in streamwise, wall-normal and spanwise directions

Case	Δ*x*/*η*	Δ*y*_*min*_/*η*	Δ*z*/*η*	Δ*x*/*η*_*B*_	Δ*y*_*min*_/*η*_*B*_	Δ*z*/*η*_*B*_
SC_1_	3.60	0.62	1.80	5.30	0.96	2.65
SC_2_	5.00	0.81	2.50	14.40	2.52	7.20
SCCTP	2.50	0.12	1.25	4.40	0.25	2.20

Only maximum values are stated

DNS data is extracted after a fully converged turbulent velocity and pressure field develops and time averaging is done using data for 10 flow through times at an interval of 2000 time steps for each of the cases mentioned above.

## Results and Discussion

### Mean flow and turbulence statistics

**Fig. 5:**
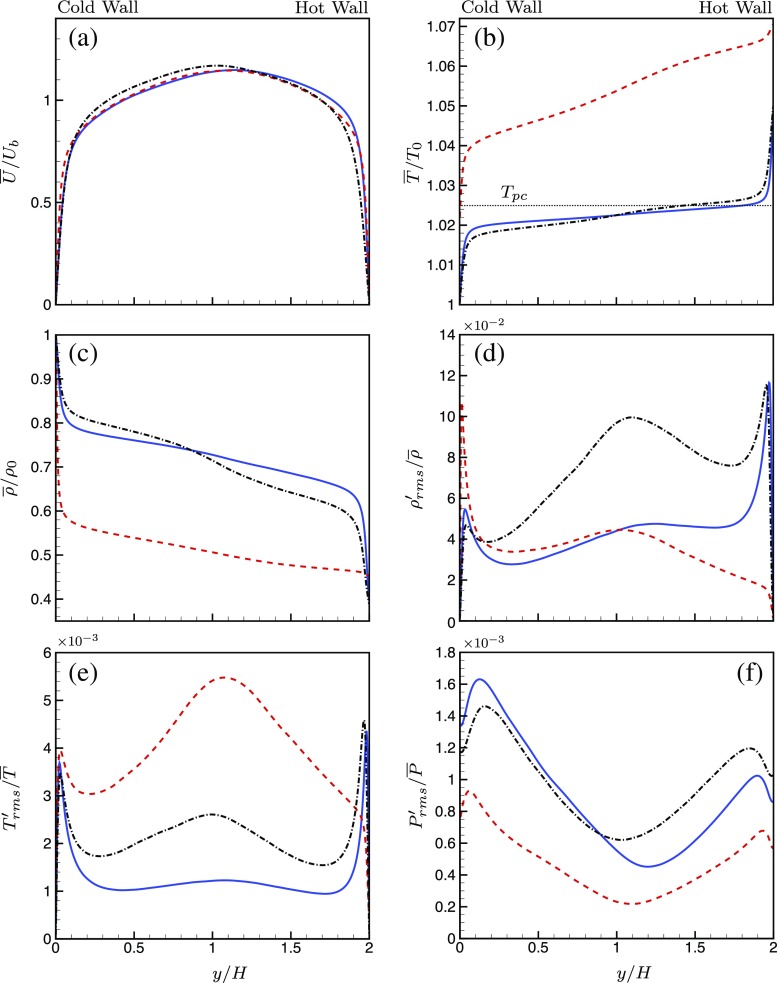
Averaged quantities as a function of channel height for **a** mean velocity, **b** mean temperature, **c** mean density, **d** density rms, **e** temperature rms, and **f** pressure rms.  SC_1_; SC_2_; SCCTP

Averaged quantities for velocity, temperature, density, density rms, temperature rms and pressure rms are shown in Fig. 5 for all three cases. The mean velocity profile for case SCCTP is almost symmetric, while this is not the case for cases SC_1_ and SC_2_, shown in Fig. 5a. This is due to the decrease in viscosity with increase in temperature, causing higher velocity gradients near the hot wall and lower velocity gradients near the cold wall.

The mean temperature profiles are presented in Fig. 5b. For cases SC_1_ and SCCTP, the trans-critical transition occurs roughly between the middle of the channel and the hot wall, which is therefore a region of high *C*_*p*_. The temperature profiles for SC_1_ and SCCTP are thus flat near the middle of the channel. On the other hand, for case SC_2_, the trans-critical transition occurs very close to the cold wall. So, the portions of the channel away from the cold wall are a region of low *C*_*p*_, causing the temperature to change more rapidly with channel height away from the cold wall. The temperature in the bulk of the channel is therefore much higher than *T*_*pc*_.

The mean density profiles are given in Fig. 5c. It can be observed that the density changes by a factor of around 2.5 from the hot wall to the cold wall. For the case SC_1_, the density changes steeply near both walls with a flatter profile near the middle of the channel. But, for the case SC_2_, the trans-critical transition very near to the cold wall results in the steep decline of density profile close to the cold wall and an approximately flat profile thereafter. It can be seen from Fig. 5b that the temperature increases more rapidly near the cold wall for the case SC_1_ compared to SCCTP. At almost the same pressure, a faster rise in temperature corresponds to a faster decline in density. Thus, the density declines at a slower rate for SCCTP compared to SC_1_. This can explain the smaller rate of increase of density near the hot wall for SCCTP. Similarly, it is seen that the temperature near the hot wall decreases less rapidly for the case SCCTP.

By observing the root mean square (rms) density fluctuation profiles for cases SC_1_ and SC_2_ in Fig. 5d, it is evident that for SC_1_, the rms density fluctuation exhibits two peaks near the walls and a smaller peak near the middle of the channel. This behaviour is analogous to turbulent flows with ideal gas with wall temperature differences. This is however not the case for SC_2_ for which the rms density fluctuation has a very high value close to the cold wall and a much smaller peak near the middle of the channel. The very high density peak close to the cold wall can be attributed to inertial effects and the effects of trans-critical transition reinforcing each other very close to the cold wall. Due to the trans-critical transition near the cold wall, the temperature for most of the remaining channel height is higher than the pseudo-critical temperature. For temperatures far away from the pseudo-critical temperature, the change of density with change in temperature is minimal. Thus, the density profile is almost flat for locations away from the cold wall. This can be seen by observing the profiles of mean density in Fig. 5c for the case SC_2_. For the other cases, SC_1_ and SCCTP, the density has sharp gradients near both walls. Due to the absence of a significant gradient of mean density near the hot wall for SC_2_, the passive mixing across a mean gradient is minimal. This causes the peak of the density fluctuation near the hot wall to disappear for SC_2_. Thus, a trans-critical transition very close to the wall causes this unusual behaviour in the density fluctuations. The rms temperature and the rms pressure fluctuations for the two cases are shown in Fig. 5e and f, respectively. The case SC_2_ has higher temperature fluctuations and lower pressure fluctuations near the center of the channel compared to the other two cases.

### Near wall turbulence

**Fig. 6:**
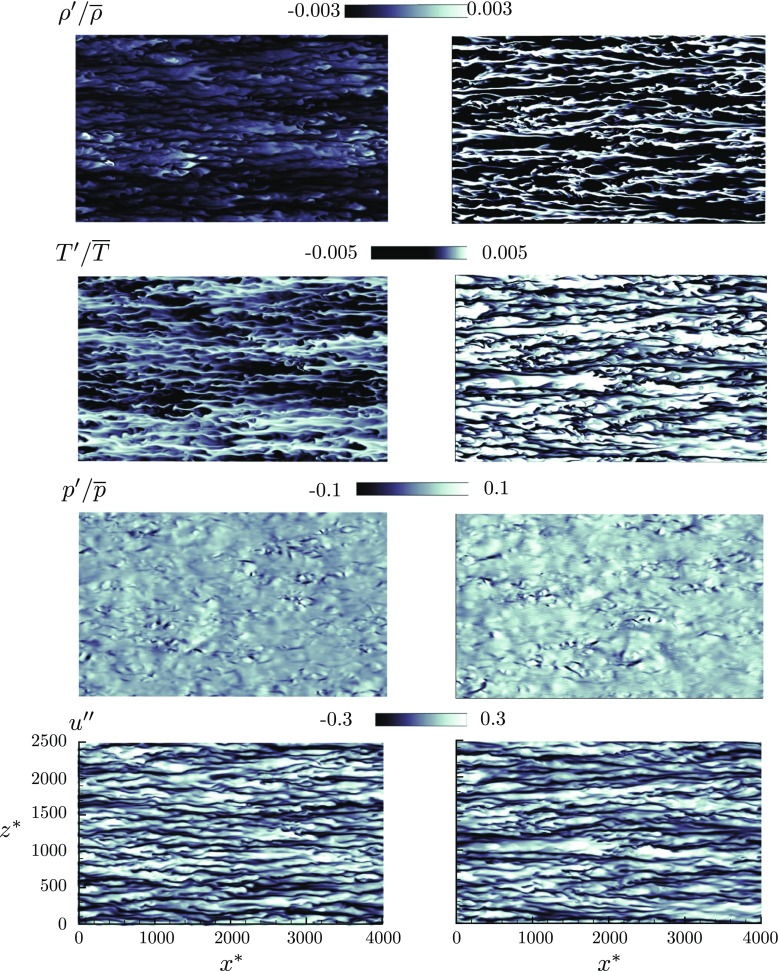
Fluctuations for streamwise velocity (*u* ^″^), density ($\rho ^{\prime } /\overline {\rho }$), temperature ($T^{\prime } / \overline {T}$), pressure ($p^{\prime } / \overline {p}$) on the hot and cold sides of the channel for turbulent flows of supercritical CO_2_ (SC_2_). From top to bottom: density streaks, temperature streaks, pressure streaks, velocity streaks. Left column - hot wall (*y* ^∗^ =14.72) and right column - cold wall (*y* ^∗^ =16.98)

The streaks for the Favre averaged streamwise velocity, density, temperature and pressure near the hot and the cold walls of the channel are shown for SC_2_ in Fig. 6. The density, pressure and temperature streaks are normalized by their local mean values. The computational box is also scaled by the semi-local scales, *x* ^∗^ and *z* ^∗^. The semi-local coordinates are defined as $x^{*}= x \overline {\rho } u_{\tau }^{*}/\overline {\mu }$ (similarly for *y* ^∗^and *z* ^∗^), with the semi-local friction velocity given as $u_{\tau }^{*} = \sqrt {\tau _{w}/\overline {\rho }}$. The streaks are calculated at the locations *y* ^∗^ =14.72near the hot wall and *y* ^∗^ =16.98near the cold wall based on the maxima for the root mean square velocity fluctuations near the respective walls. For this case, it is seen that there is no peak for the density fluctuation near the hot wall. This is due to the trans-critical transition very close to the cold wall. This behaviour is affirmed on analyzing the density fluctuation in Fig. 5d, when it is seen that near the hot wall there are almost no high density streaks, whereas near the cold wall high density streaks are more prevalent. The temperature streaks indicate the same behaviour to a lesser degree. Near the cold wall, there is a higher occurrence of high temperature fluctuations compared to that near the hot wall. The streaks also prove that, near the walls, the density is much more correlated with temperature compared to pressure, since no significant occurrences of high or low pressure fluctuations can be seen near the walls. The analysis of Favre averaged velocity fluctuations near the walls reveal that the high speed streaks are more enhanced near the cold wall compared to the hot wall. Also, the velocity streaks indicate more coherent structures near the cold wall.

### Mean velocity transformations

**Fig. 7:**
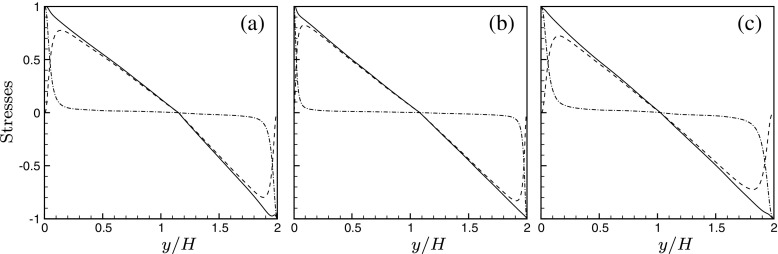
Shear stresses as a function of channel height.  $-\overline {\rho }\overline {u^{\prime }v^{\prime }} / \tau _{w}$;  $\left (\overline {\mu } \partial \overline {U} / \partial y\right ) / \tau _{w}$;  $\left (-\overline {\rho }\overline {u^{\prime }v^{\prime }} + \left (\overline {\mu } \partial \overline {U} / \partial y\right )\right ) / \tau _{w}$. **a** SC_1_; **b** SC_2_; **c** SCCTP

For all of the above mentioned simulations, the walls are at different temperatures and the wall stresses at the cold and hot walls are different from one another. Therefore, the channel has been divided into hot and cold sides depending on the location of zero Reynolds shear stress. The locations of zero shear stress occur at *y*/*H* =1.1488,1.0765,1.0172 for the cases SC_1_, SC_2_and SCCTP, respectively. The laminar, turbulent and total shear stress on the hot and the cold sides of the channel, scaled by the wall stress on the respective sides, are shown in Fig. 7. The total shear stress shows a kink near the channel centre due to the loss of symmetry.

**Fig. 8:**
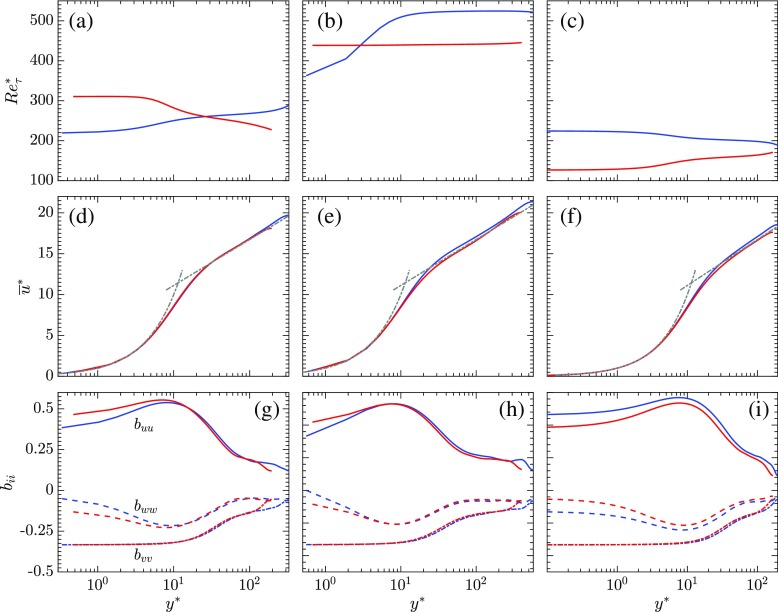
*R* *e* *τ*∗(top row), extended van Driest transformed velocity (middle row), and turbulence anisotropies (bottom row) as a function of *y* ^∗^; (left column) SC_1_, (middle column) SC_2_, (right column) SCCTP;  cold side, hot side

Based on [26], the changes in the semi-local Reynolds number, $Re_{\tau }^{*}$, defined as $Re_{\tau }^{*} = \overline {\rho } u_{\tau }^{*} H/\overline {\mu } = \sqrt {\overline {\rho } / \rho _{w}} / \left (\overline {\mu } / \mu _{w}\right )Re_{\tau w}$, have a significant impact on inter-component energy transfer and hence on turbulence anisotropy in the flow field. The anisotropies are defined as $b_{ij} = \overline {u_{i}^{\prime }u_{j}^{\prime }} / \overline {u_{k}^{\prime }u_{k}^{\prime }} - 1/3\delta _{ij} $, where *δ*_*ij*_ is the Kronecker delta. When $dRe_{\tau }^{*} / dy < 0$, it causes a reduction in momentum transfer and redistribution in turbulent kinetic energy from the streamwise direction to the other directions. This is evident in an increase in the streamwise anisotropy and a decrease in the spanwise anisotropy. The opposite behaviour is observed when $dRe_{\tau }^{*} / dy > 0$. The semi-local Reynolds number $Re_{\tau }^{*}$ and the anisotropies *b*_*uu*_, *b*_*ww*_ and *b*_*vv*_in the streamwise, spanwise and wall-normal directions, respectively, are show for the three simulations in Fig. 8. It is seen from Fig. 8a and g, that for SC_1_, the streamwise anisotropy on the cold side is less than that on the hot side of the channel. This can be explained by the fact that $dRe_{\tau }^{*} / dy > 0$ on the cold side of the channel and $dRe_{\tau }^{*} / dy < 0$ on the hot side of the channel. So, there is a reduction in redistribution of turbulent kinetic energy on the hot side and an increase on the cold side of the channel. The opposite is observed for the streamwise anisotropy in SCCTP as $Re_{\tau }^{*}$ is decreasing on the cold side of the channel and increasing on the hot side of the channel. This is demonstrated in Fig. 8c and i. From Fig. 8b and h, it is observed that, for SC_2_, the anisotropies are nearly equal on the two sides of the channel due to the flatter $Re_{\tau }^{*}$ profiles as a result of which $dRe_{\tau }^{*} / dy$ is almost equal to zero for most of the channel height. For all the cases, it is seen that an increase in streamwise anisotropy is accompanied with a corresponding decrease in the spanwise anisotropy, while the anisotropy in the wall-normal direction in the hot and cold sides of the channel seems to be relatively unaffected, whether $Re_{\tau }^{*}$ is increasing or decreasing. The van Driest transformation has been previously used to scale the streamwise velocity $\left (\overline {u}^{VD}\right )$ in compressible flows. This provides a reasonable collapse of the velocity profiles with the log law of the wall. A more recent approach is the extended van Driest transformation developed by [27] and [28]. The extended van Driest transformed velocity ($\overline {u^{*}}$) is plotted as a function of *y* ^∗^for the three simulations in Fig. 8d, e and f. This is seen to produce a fairly good collapse of the velocity profiles with the log law for the wall.

### Semi locally scaled turbulent statistics and budgets

The velocity fluctuations and the quantities involved in evaluating the budgets for the turbulent kinetic energy ($k = 1/2 \overline {\rho } \widetilde {u_{i}^{\prime \prime }u_{i}^{\prime \prime }}$) are scaled by the semi-local variables and plotted as a function of *y* ^∗^. The budget equation used is the same as that given by [29] and [30], and is given as follows,

22 $$ P_{k} + D_{k} + \epsilon_{k} + C_{k} =0, $$

with the turbulent production *P*_*k*_, diffusion *D*_*k*_, dissipation *𝜖*_*k*_ and compressibility terms *C*_*k*_. These are expressed as

23 $$ P_{k} = -\overline{\rho} \widetilde{u^{\prime\prime}v^{\prime\prime}} \frac{\partial \widetilde{u}}{ \partial y}; D_{k} = \frac{\partial \left[ \overline{{\Gamma}_{i2}^{\prime}u_{i}^{\prime}} - \overline{\rho} \widetilde{v^{\prime\prime}u_{i}^{\prime\prime}u_{i}^{\prime\prime}} - \overline{p^{\prime}v^{\prime}}\right]}{\partial y} $$

24 $$ \epsilon_{k} = -\overline{{\Gamma}_{ij}^{\prime}\frac{\partial u_{i}^{\prime}}{\partial x_{j}}}; C_{k} = - C_{k1} + C_{k2} + C_{k3} $$

25 $$ C_{k1} = \overline{v^{\prime\prime}}\frac{\partial \overline{p}}{\partial y}; C_{k2} = \overline{u_{i}^{\prime\prime}\frac{\partial {\Gamma}_{i2}}{\partial y}}; C_{k3} = \overline{p^{\prime}\frac{\partial u_{k}^{\prime}}{\partial x_{k}}}. $$

**Fig. 9:**
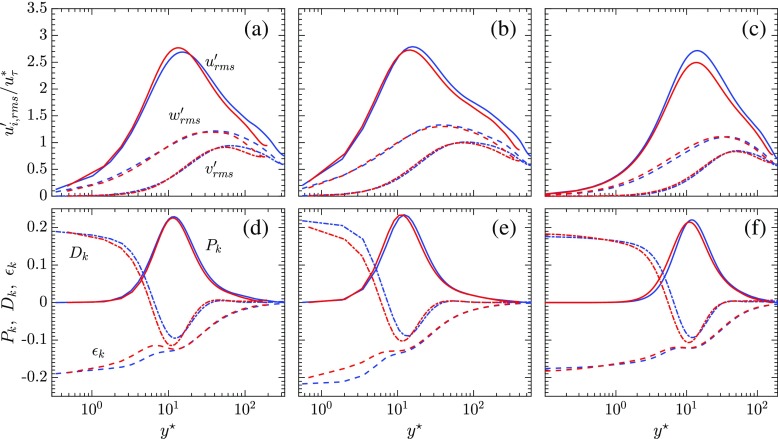
Semi-locally scaled velocity fluctuations (top row) and turbulent kinetic energy budgets (bottom row) as a function of *y* ^∗^. (left column) SC_1_, (middle column) SC_2_, (right column) SCCTP;  cold side, hot side

The velocity fluctuations and the turbulent kinetic energy budgets scaled by the semi-local variables are shown for the three cases in Fig. 9. The velocity fluctuations and the turbulent kinetic energy budgets, if scaled by bulk variables do not collapse on top of each other. However, the values on the hot and cold sides of the channel are found to collapse when scaled by semi-local variables. The validity of the semi-local scaling also reaffirms the relevance of the Morkovin hypothesis for all of the simulations performed. This signifies that the changes in the turbulence structures are due to changes in mean density and mean viscosity gradients and fluctuations of these quantities have a limited impact on the turbulence in the flow.

### Inertial and viscous effects

As the flow is compressible in nature with variable viscosity, the turbulence in the flow is influenced both by the variable inertial and viscous effects on the hot and cold side of the channel. The magnitudes of the inertial and viscous forces on the hot and cold sides of the channel were quantified by [25]. These are given as follows,

26 $$ {F_{I}^{h}} \propto {\rho_{b}^{h}} {U_{b}^{h}} (h^{h})^{2};{F_{I}^{c}} \propto {\rho_{b}^{c}} {U_{b}^{c}} (h^{c})^{2} $$

where (${F_{I}^{h}}$ ${F_{I}^{c}}$), (${\rho _{b}^{h}}$ ${\rho _{b}^{c}}$), (${U_{b}^{h}}$ ${U_{b}^{c}}$) and (*h* ^*h*^ *h* ^*c*^) refer to the inertial force, the bulk density, the bulk velocity and the channel height on the hot and cold sides of the channel, respectively.

27 $$ {F_{V}^{h}} \propto {\mu_{b}^{h}}{U_{b}^{h}}h^{h};{F_{V}^{c}} \propto {\mu_{b}^{c}}{U_{b}^{c}}h^{c} $$

where (${F_{V}^{h}}$ ${F_{V}^{c}}$) and (${\mu _{b}^{h}}$ ${\mu _{b}^{c}}$) refer to the viscous forces and the bulk viscosity on the hot and cold sides of the channel, respectively. The ratio of inertial and viscous forces on the hot and cold sides of the channel are given in Table 4.

**Table 4:** Ratio of the inertial and viscous forces on the hot and cold sides of the channel

Case	${F_{I}^{c}} / {F_{I}^{h}}$	${F_{V}^{c}} / {F_{V}^{h}}$
SC_1_	2.03	1.60
SC_2_	1.57	1.23
SCCTP	1.39	1.06

This shows that in all the cases, there is an increase in the viscous forces from the hot to the cold side of the channel, but there is an even bigger increase in inertial forces. This is due to the fact that the inertial force is proportional to the square of the channel height and the channel height on the cold side is greater than the channel height on the hot side for each of the cases simulated. Also, for the constant viscosity case, the ratio of the viscous forces on the two sides of the channel is found to be close to unity. This is not the case with the variable viscosity cases.

### Compressibility effects

**Fig. 10:**
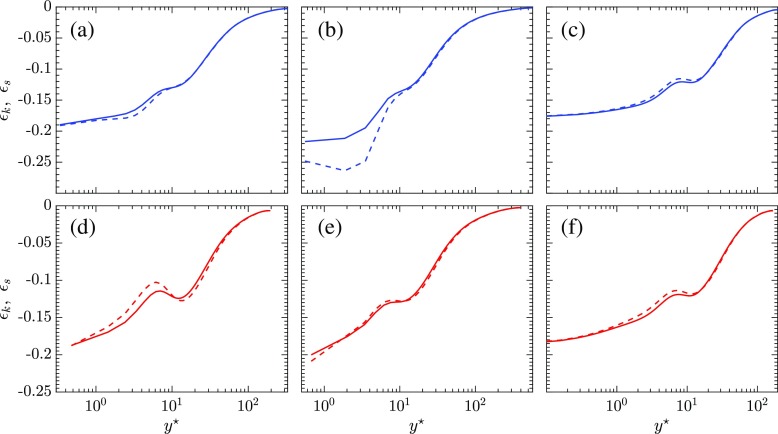
Total *𝜖*_*k*_  and solenoidal *𝜖*_*s*_ dissipation as a function of *y* ^∗^; (left column) SC_1_, (middle column) SC_2_, (right column) SCCTP; cold side, hot side

Previous authors, such as [30, 31] have divided the turbulent kinetic energy dissipation into the sum of the solenoidal, dilatational and inhomogeneous parts. The solenoidal dissipation ($\epsilon _{s} = - \overline {\mu }\overline {\omega _{ij}^{\prime } \omega _{ij}^{\prime }}$) represents the pseudo-dissipation for the corresponding incompressible flow, where *ω* is used to refer to the vorticity in the flow field. The inhomogeneous component of the dissipation is found to be neglible. The dilatational dissipation is the part of the total dissipation attributed to compressibility effects. So, the total dissipation can be divided into the sum of the incompressible and compressible parts, namely, the solenoidal and the dilatational dissipation. The compressibility effects are thereby evaluated by comparing the solenoidal dissipation to the total dissipation for turbulent kinetic energy (*𝜖*_*k*_). Both of these quantities on the hot and cold sides of the channel are scaled by the semi-local variables and are plotted in Fig. 10. If compressibility effects are minimal, the solenoidal dissipation and the total dissipation should almost collapse on top of each other. The biggest difference between the solenoidal and the total dissipation is observed in the case SC_2_ very near to the cold wall. The other cases SC_1_ and SCCTP with trans-critical transition near the middle of the channel do not exhibit significant levels of compressibility close to the walls as the solenoidal and total dissipations almost collapse on top of each other. So, for turbulent flows of supercritical CO_2_, it is possible to experience significant levels of compressibility caused by trans-critical transition near to the cold wall. But, overall, the compressibility effects experienced are still small as they are significant only in the case of trans-critical transition very close to the wall and are confined to a limited region close to the wall. It is expected that these effects will be more pronounced for higher Mach number cases or for pressures closer to the critical pressure.

## Conclusion

Fully compressible direct numerical simulations are performed for turbulent flows of supercritical CO_2_ at low Mach numbers close to the vapour-liquid critical point involving trans-critical transition. The main conclusions from our research are as follows.

The location of trans-critical transition has an influence on the mean and turbulent statistics. Comparison of the mean temperature and the mean density profile for the cases SC_1_ and SC_2_, reveal significant differences. These are due to the changing nature of the density and the isobaric heat capacity for CO_2_. When the pseudo-critical temperature is located very close to the cold wall, as for the case SC_2_, it is seen to alter the traditional nature of turbulence. This is seen in the unusual nature of the density fluctuations, which is not observed in turbulent ideal gas flows with a wall temperature difference. The analysis of the streaks for density and temperature for SC_2_ also indicate that the cold wall region is characterized by much higher occurrences of high temperature and high density fluctuations than the region near the hot wall. The streamwise velocity streaks for SC_2_ near the hot and cold walls signify that high speed streaks are much more prevalent near the cold wall and also, the structures near the cold wall have a higher degree of coherence. A comparison of the semi-locally scaled solenoidal and total dissipation indicates that trans-critical transition very close to the wall can produce compressibility effects, even at low Mach numbers. This is observed from the significant differences between solenoidal and total dissipation close to the cold wall for SC_2_. But, the compressibility effects in the overall perspective are quite small as these are only confined to a small part of the channel very close to the cold wall and also, for all the other simulations, they almost collapse on top of each other. The compressibility effects should become even more accentuated for higher Mach number cases.

The change in the nature of the $Re_{\tau }^{*}$ profiles is has a significant impact on the redistribution of turbulent kinetic energy. This is evident in the fact that, on comparing the streamwise anisotropies on the hot and cold sides of the channel, the anisotropy is seen to increase when $dRe_{\tau }^{*} / dy < 0$ due to which there is a reduction in the redistribution of turbulent kinetic energy form the streamwise to the other directions. The opposite behaviour is observed when $dRe_{\tau }^{*} / dy > 0$. Thus, we can conclude that liquid-like behaviour which is characterized by an increasing $Re_{\tau }^{*}$ causes a decrease in the streamwise anisotropy and an increase in the spanwise anisotropy due to the increase in the turbulent kinetic energy redistribution from streamwise to the other directions, and the opposite is seen with gas-like behaviour.

The semi-locally scaled velocity fluctuations and turbulent kinetic energy budgets on the hot and cold sides of the channel are found to collapse almost on top of each other. This result is seen to reinforce Morkovin’s hypothesis as it establishes that the changes in the turbulence structure are only caused by the change in the mean density gradients in the flow and the influence of density fluctuations on the changes in turbulence structures is insignificant.

## Figures and Tables

**Fig. 11:**
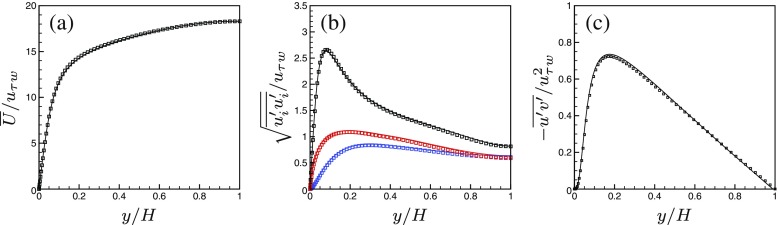
Validation of the compressible DNS code [22]; Lines: present; Symbols: KMM

**Fig. 12:**
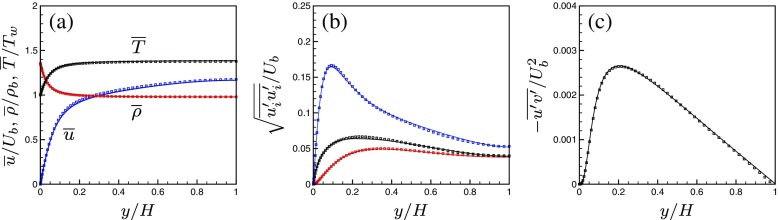
Validation of the compressible DNS code [23]; Lines: present; Symbols: CKM

## Appendix

### Appendix A: Derivation of departure functions

The non-dimensional van der Waals (VdW) equation of state is given as:

28 $$ P = \frac{RT}{\vartheta - b} - \frac{a}{\vartheta^{2}}, $$

where *P* is the pressure, *T* is the temperature, *𝜗* is the specific volume. The non-dimensionalization is performed as follows:

29 $$ R=\frac{R^{\star} T_{0}^{\star}}{U_{0}^{\star 2}};a=\frac{a^{\star} \rho_{0}^{\star}}{U_{0}^{\star 2}};b=b^{\star}\rho_{0}^{\star}. $$

The dimensional constants *a* ^⋆^and *b* ^⋆^are determined by the critical properties of the real gas. These constants are given as:

30 $$ a^{\star} =3P_{c}^{\star}\left( V_{c}^{\star 2}\right);b^{\star} = V_{c}^{\star}/3, $$

where $P_{c}^{\star }$ is the critical pressure and $V_{c}^{\star }$ is the critical volume of the gas. The residual internal energy (*U* ^*R*^) is given as:

31 $$ U^{R} = U - U^{ig} = \int\limits_{\infty}^{\vartheta}\left( T\left( \frac{\partial S}{\partial \vartheta}\right)_{T} - P\right)d\vartheta. $$

Using Maxwell’s relations, we get: (*∂* *S*/*∂* *𝜗*)_*T*_ =(*∂* *P*/*∂* *T*)_*𝜗*_. Substituting in the integral, $U^{R} = \int \limits _{\infty }^{\vartheta }\left (T\left (\partial P / \partial T\right )_{\vartheta } - P\right )d\vartheta $. Furthermore, from the van der Waals equation: (*∂* *P*/*∂* *T*)_*𝜗*_ = *R*/ (*𝜗* − *b*). The expression for the residual internal energy is thereby given as: $U^{R} = \int \limits _{\infty }^{\vartheta } \left (a / \vartheta ^{2}\right ) d\vartheta $. This has also been given in the works published by previous authors, such as [32, 33]. Thus, we get:

32 $$ U - C_{\vartheta}T = -\frac{a}{\vartheta}; U = C_{\vartheta}T - a\rho, $$

where *C*_*𝜗*_is the heat capacity of the gas at constant volume.

The non-dimensional Redlich Kwong (RK) equation of state is given as:

33 $$ P = \frac{RT}{\vartheta - b} - \frac{a\alpha}{\left( \vartheta\right)\left( \vartheta+b\right)}. $$

The dimensional constants *a* ^⋆^and *b* ^⋆^ are given as: $a^{\star }= \left (0.42748 R^{\star 2} T_{c}^{\star 2.5}\right ) / \left (P_{c}\right )$, $b^{\star }=\left (0.08662R^{\star }T_{c}^{\star }\right ) / \left (P_{c}^{\star }\right )$. The parameter *α* is given as: $\alpha =1 / \sqrt {T^{\star }}$.

For this equation of state, following the same procedure as mentioned above, we get,

34 $$ T\left( \frac{\partial P}{\partial T}\right)_{\vartheta} - P = \frac{a\left( \alpha - T\frac{d \alpha}{d T}\right)}{\left( \vartheta\right)\left( \vartheta+b\right)}. $$

So, the residual internal energy is given as:

35 $$ U^{R} = \int\limits_{\infty}^{\vartheta}\left( T\left( \frac{\partial P}{\partial T}\right)_{\vartheta} - P\right) = \frac{a\left( \alpha - T\frac{d \alpha}{d T}\right)}{b} log \frac{\vartheta}{\vartheta+b}. $$

Substituting the value of *α*

36 $$ U = C_{\vartheta}T + \frac{3}{2}\frac{a}{b\sqrt{T}} log \frac{1}{1+b\rho}. $$

### Appendix B: Validation

The compressible Navier-Stokes code has been validated with the data published by previous authors for both low Mach number [22] and high Mach number [23] cases. This is shown in the Figs. 11 and 12 with the help of both mean and turbulent statistics. The low Mach number and high Mach number cases used for validating the code are henceforth referred to as KMM and CKM, respectively. The corresponding simulations carried out with our present code are called Ma0.2 and Ma1.5. The details of these simulations are given in Table 5. The plots in Figs. 11 and 12 show a fairly good agreement for the mean flow profiles, the turbulent velocity fluctuations and the Reynolds shear stress with the data published by the previous authors.

**Table 5:** Validation of the code for low and high Mach number cases using ideal gas equation of state; *M* *a*: Mach number; *R* *e*: bulk Reynolds number; *R* *e*_*τ*_: friction Reynolds number at the wall; *P* *r*: Prandtl number; *N*_*x*_,*N*_*y*_,*N*_*z*_: number of grid points; *L*_*x*_,*L*_*y*_,*L*_*z*_: lengths of channel

Case	*M* *a*	*R* *e*	*R* *e*_*τ*_	*P* *r*	*N*_*x*_ × *N*_*y*_ × *N*_*z*_	*L*_*x*_ × *L*_*y*_ × *L*_*z*_
KMM	0.0	2800	178	0.7	192 × 129 × 160	$4\pi h \times 2h \times \frac {4}{3}\pi h$
CKM	1.5	3000	222	0.7	144 × 119 × 80	$4\pi h \times 2h \times \frac {4}{3}\pi h$
Ma0.2	0.2	2800	180	0.7	120 × 168 × 120	4*π* *h* × 2*h* × 2*π* *h*
Ma1.5	1.5	3000	209	0.7	120 × 168 × 120	4*π* *h* × 2*h* × 2*π* *h*
